# Assessment of the Accuracy of Using *ICD-9* Diagnosis Codes to Identify Pneumonia Etiology in Patients Hospitalized With Pneumonia

**DOI:** 10.1001/jamanetworkopen.2020.7750

**Published:** 2020-07-22

**Authors:** Thomas L. Higgins, Abhishek Deshpande, Marya D. Zilberberg, Peter K. Lindenauer, Peter B. Imrey, Pei-Chun Yu, Sarah D. Haessler, Sandra S. Richter, Michael B. Rothberg

**Affiliations:** 1 The Center for Case Management, Natick, Massachusetts; 2 Department of Critical Care Medicine, University of Massachusetts Medical School-Baystate, Springfield; 3 Center for Value-Based Care Research, Cleveland Clinic Community Care, Cleveland Clinic, Cleveland, Ohio; 4 EviMed Research Group LLC, Goshen, Massachusetts; 5 Institute for Healthcare Delivery and Population Science, Department of Medicine, University of Massachusetts Medical School-Baystate, Springfield; 6 Department of Quantitative Health Sciences, University of Massachusetts Medical School, Worcester; 7 Department of Quantitative Health Sciences, Lerner Research Institute, Cleveland Clinic, Cleveland, Ohio; 8 Cleveland Clinic, Lerner College of Medicine, Case Western Reserve University, Cleveland, Ohio; 9 Division of Infectious Diseases, Department of Medicine, University of Massachusetts Medical School-Baystate, Springfield; 10 Department of Laboratory Medicine, Cleveland Clinic, Cleveland, Ohio

## Abstract

**Importance:**

Administrative databases may offer efficient clinical data collection for studying epidemiology, outcomes, and temporal trends in health care delivery. However, such data have seldom been validated against microbiological laboratory results.

**Objective:**

To assess the validity of *International Classification of Diseases, Ninth Revision (ICD-9)* organism-specific administrative codes for pneumonia using microbiological data (test results for blood or respiratory culture, urinary antigen, or polymerase chain reaction) as the criterion standard.

**Design, Setting, and Participants:**

Cross-sectional diagnostic accuracy study conducted between February 2017 and June 2019 using data from 178 US hospitals in the Premier Healthcare Database. Patients were aged 18 years or older admitted with pneumonia and discharged between July 1, 2010, and June 30, 2015. Data were analyzed from February 14, 2017, to June 27, 2019.

**Exposures:**

Organism-specific pneumonia identified from *ICD-9* codes.

**Main Outcomes and Measures:**

Sensitivity, specificity, positive predictive value, and negative predictive value of *ICD-9* codes using microbiological data as the criterion standard.

**Results:**

Of 161 529 patients meeting inclusion criteria (mean [SD] age, 69.5 [16.2] years; 51.2% women), 35 759 (22.1%) had an identified pathogen. *ICD-9*–coded organisms and laboratory findings differed notably: for example, *ICD-9* codes identified only 14.2% and 17.3% of patients with laboratory-detected methicillin-sensitive *Staphylococcus aureus* and *Escherichia coli*, respectively. Although specificities and negative predictive values exceeded 95% for all codes, sensitivities ranged downward from 95.9% (95% CI, 95.3%-96.5%) for influenza virus to 14.0% (95% CI, 8.8%-20.8%) for parainfluenza virus, and positive predictive values ranged downward from 91.1% (95% CI, 89.5%-92.6%) for *Staphylococcus aureus* to 57.1% (95% CI, 39.4%-73.7%) for parainfluenza virus.

**Conclusions and Relevance:**

In this study, *ICD-9* codes did not reliably capture pneumonia etiology identified by laboratory testing; because of the high specificities of *ICD-9* codes, however, administrative data may be useful in identifying risk factors for resistant organisms. The low sensitivities of the diagnosis codes may limit the validity of organism-specific pneumonia prevalence estimates derived from administrative data.

## Key Points

**Question:**

Are organism-specific *International Classification of Diseases, Ninth Revision (ICD-9)* administrative codes for pneumonia valid measures in identifying pneumonia etiology?

**Findings:**

In this cross-sectional study of data from 161 529 patients hospitalized with pneumonia between 2010 and 2015, *ICD-9* codes had generally low sensitivity but high specificity for pneumonia etiology identified by laboratory testing.

**Meaning:**

In this study, *ICD-9* codes appeared to underestimate prevalence of specific organisms.

## Teaser

This cohort study assesses the validity of organism-specific *International Classification of Diseases, Ninth Revision (ICD-9)* administrative codes for pneumonia in adult patients using microbiological data as the criterion standard.

## Introduction

Although detailed clinical data represent the criterion standard for studying epidemiology, outcomes, and temporal trends in health care delivery, such data are cumbersome and expensive to collect. It is difficult to create research data sets large enough to represent the patient mix and the variety of health care settings; medical record abstraction requires intensive review by trained professionals and is subject to interobserver variability and observer bias. The Centers for Disease Control and Prevention directs surveillance of specific health care–associated infections captured by the National Hospital Surveillance Network and engages a small number of academic centers to collect data through the Centers for Disease Control and Prevention Epicenters Program, but these data are limited in scope.^1,2^ In contrast, administrative data collected during routine clinical encounters for the purpose of reimbursement are copious, widely available, and generalizable. For these reasons, administrative data offer a potential alternative for some types of research. Administrative data have been used, for example, to evaluate temporal trends in pneumonia hospitalization and mortality, but there remains a paucity of efforts to validate administrative data with corresponding clinical information.^3^ Administrative data can be imprecise, with claims-based algorithms for some conditions demonstrating lower mortality, length of stay, and costs than independent clinical review.^4^

Validation studies testing the accuracy of pathogen-specific coding have been rare in hospitalizations for infectious diseases in general and in pneumonia in particular. To establish the validity of administrative data regarding pneumonia, we examined the performance of pathogen-specific administrative coding in comparison with corresponding microbiological data in the setting of community-onset pneumonia in a large multicenter US database.

## Methods

In this cross-sectional diagnostic accuracy study, we studied patients hospitalized with pneumonia between July 1, 2010, and June 30, 2015, using data from 178 US hospitals in the Premier Healthcare Database. Data were analyzed from February 14, 2017, to June 27, 2019. Using microbiological evidence of a pathogen as the criterion standard (test results for blood or respiratory culture, urinary antigen, or polymerase chain reaction), we derived the performance characteristics (sensitivity, specificity, positive predictive value [PPV], and negative predictive value [NPV]) of the corresponding *ICD-9* organism codes as indicators of diagnosis. Because the data source was completely deidentified, the institutional review board of the Cleveland Clinic determined that this study was exempt from review and did not require informed patient consent. This study followed the Standards for Reporting of Diagnostic Accuracy (STARD) reporting guideline for diagnostic accuracy studies.

The Premier Healthcare Database is widely used for research and has been well described elsewhere.^5^ Between July 1, 2010, and June 30, 2015, the number of participating hospitals increased from 461 to 592. In 2015, 75% of participating hospitals were in urban settings (census block groups or blocks have a population density of at least 1000 people per square mile) and 25% were rural by the US Census Bureau definition (any territory outside urban setting),^6^ mirroring the membership of the American Hospital Association, although with Midwestern hospitals underrepresented and Southern hospitals overrepresented. Larger hospitals were overrepresented and teaching hospitals were underrepresented in the Premier Healthcare Database. For the current analysis, we included the 178 hospitals in the Premier Healthcare Database that reported microbiological data using the Safety Surveillor web-based tracking tool.

We included all patients aged 18 years or older who were discharged between July 1, 2010, and June 30, 2015, with either a principal diagnosis of pneumonia or with a principal diagnosis of respiratory failure, acute respiratory distress syndrome, respiratory arrest, sepsis, or influenza and a secondary diagnosis of pneumonia (details of the algorithm have been published previously).^7^ In addition, a blood culture, respiratory culture, pneumococcal urinary antigen, *Legionella* urinary antigen, or antibody tests or polymerase chain reaction targeting atypical bacterial pathogens (*Bordetella pertussis*, *Chlamydophila pneumonia*e, *Mycoplasma pneumoniae)* or viruses was required for inclusion. Included tests are listed in the eTable in the Supplement. Patients with a secondary diagnosis of cellulitis, cholecystitis, appendicitis, diverticulitis, perforated diverticulum, peritonitis, postoperative anastomotic leaks, or abdominal surgical site infections were excluded (Figure).

**Figure.**
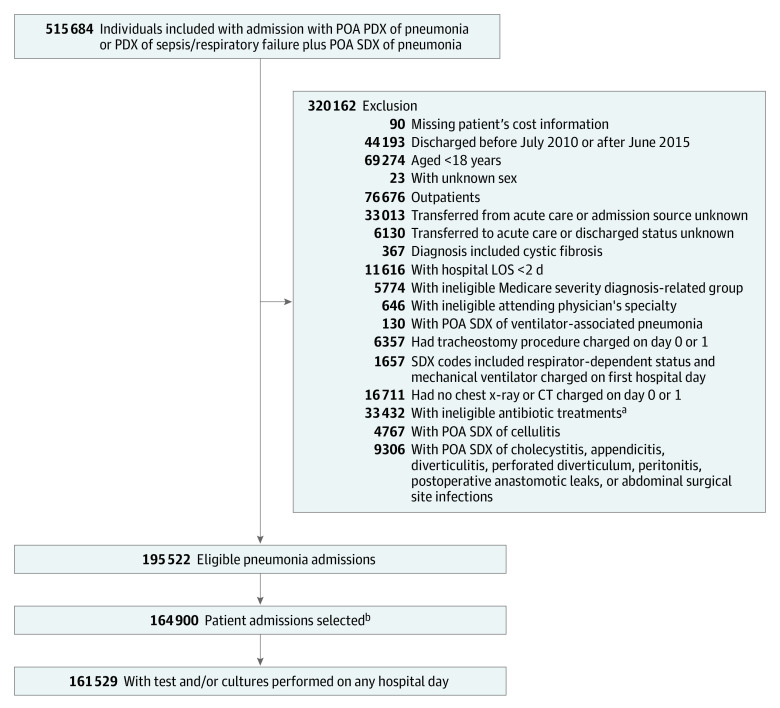
Participant Selection Flow Diagram Abbreviations: CT, computed tomography; LOS, length of stay (in days); PDX, principal diagnosis; POA, present on admission; SDX, secondary diagnosis. ^a^Patients with viral pneumonia as principal diagnosis but without initial antibiotic treatments were included. ^b^On a 1-patient 1-admission basis, with a single eligible admission randomly selected from each patient's eligible admissions (178 hospitals).

### Statistical Analysis

Baseline characteristics of patients with pneumonia were summarized by frequency distributions for categorical variables and mean (SD) or quartiles for continuous variables. We cross-classified the presence vs absence of an *ICD-9* code for each of the 12 common organisms identified as causing pneumonia in hospitalized patients with the presence or absence of a laboratory sample identifying that organism. From these cross-classifications, we calculated 4 measures of *ICD-9* code performance in designating a laboratory-confirmed organism: (1) sensitivity, the fraction of patients with a positive laboratory finding of an organism for whom the *ICD-9* code of that organism was present; (2) specificity, the fraction of patients without a positive laboratory finding for an organism for whom the *ICD-9* code of that organism was also not present; (3) PPV, the fraction of patients with an *ICD-9* code of an organism for whom a corresponding laboratory finding was present; and (4) NPV, the fraction of patients without an *ICD-9* code for an organism who were also without a laboratory finding for the organism. For the 4 most common organisms, sensitivity, specificity, PPVs, and NPVs were also tracked across year and by whether the diagnosis was primary or secondary. For any particular laboratory test, patients without a result were assumed not to have had that test. Data management and analysis were performed with SAS statistical software version 9.4 (SAS Institute).

## Results

The database included 515 684 patients before exclusions (Figure); among the 164 900 patients admitted with pneumonia who met inclusion criteria, cultures were obtained from 161 529 (98.0%) (mean [SD] age, 69.5 [16.2] years; 51.2% women) (Table 1), including blood cultures from 154 034 (93.4%) patients. Most patients (71.8%) were insured by Medicare, 87.8% were admitted through the emergency department, and most had a principal diagnosis of pneumonia (61.9%, including 9.3% by aspiration) or sepsis (32.1%). One-quarter of patients were treated in the intensive care unit, and 8.4% received invasive mechanical ventilation. The in-hospital mortality rate was 9.2%, and median length of stay was 5 days (interquartile range, 3-8 days). Median cost was $8356.41 (interquartile range, $5035.31-$14 928.49). Of the entire eligible cohort, 35 759 (22.1%) had a positive test result.

**Table 1.** Characteristics of 161 529 Patients Hospitalized With Pneumonia in the 2010-2015 Premier Healthcare Database Sample

Characteristic	No. (%)
Age, mean (SD), y	69.5 (16.2)
Male	78 786 (48.8)
Race/ethnicity	
White	124 574 (77.1)
Black	20 348 (12.6)
Other	16 607 (10.3)
Admission source	
Emergency department	141 861 (87.8)
SNF/ICF	12 431 (7.7)
Clinic	7100 (4.4)
Other	137 (0.08)
Discharge status	
Home	95 782 (59.3)
Hospice	8534 (5.3)
Died	14 798 (9.2)
SNF	38 957 (24.1)
Other	3458 (2.1)
Insurance	
Medicare	116 020 (71.8)
Medicaid	14 059 (8.7)
Commercial/managed care	22 312 (13.8)
Other	9138 (5.7)
Principal diagnosis code	
Viral pneumonia	63 (0.04)
Pneumonia	81 359 (50.4)
Aspiration pneumonia	15 021 (9.3)
Influenza pneumonia	3499 (2.2)
Sepsis	51 823 (32.1)
Respiratory failure	9764 (6.0)
Teaching hospital	66 317 (41.1)
Urban setting (US Census Bureau definition)^a^	142 356 (88.1)
Beds, No.	
≤200	33 145 (20.5)
201-400	69 184 (42.8)
≥401	59 200 (36.6)
Region (US Census Bureau classification)^8^	
Midwest	40 664 (25.2)
Northeast	30 183 (18.7)
South	69 041 (42.7)
West	21 641 (13.4)
Combined comorbidity score, mean (SD)^b^	3.2 (2.7)
Hypertension	106 492 (65.9)
Fluid and electrolyte disorders	80 617 (49.9)
Chronic pulmonary disease	75 327 (46.6)
Anemia	53 072 (32.9)
Diabetes	52 827 (32.7)
Congestive heart failure	45 295 (28.0)
Other neurological disorders	27 301 (16.9)
Hypothyroidism	27 452 (17.0)
Depression	25 379 (15.7)
Weight loss	20 720 (12.8)
Obesity	21 797 (13.5)
Valvular disease	15 392 (9.5)
Coagulopathy	15 309 (9.5)
Peripheral vascular disease	13 204 (8.2)
Pulmonary circulation disease	12 820 (7.9)
Psychoses	10 130 (6.3)
Admit from SNF	12 431 (7.7)
Previous admission (within 6 mo)	17 395 (10.8)
Immunosuppressed	21 638 (13.4)
ICU	41 226 (25.5)
IMV	13 572 (8.4)
Vasopressor	11 413 (7.1)
In-hospital mortality	14 798 (9.2)
Length of stay, median (IQR), d	5.0 (3.0-8.0)
Cost, median (IQR), $	8356.41 (5035.31-14 928.49)

Abbreviations: ICF, intermediate care facility; ICU, intensive care unit; IMV, invasive mechanical ventilation; IQR, interquartile range; SNF, skilled nursing facility.

^a^ Urban setting indicates census block groups or blocks with a population density of at least 1000 people per square mile.^6^

^b^ Theoretical range, −2 (lowest mortality risk) to 20 (highest mortality risk); range in these data, −1 to 19.

Most patients (110 360 [68.3%]) had an *ICD-9* code for pneumonia, organism unspecified (486). The organisms most frequently specified were influenza (5891 [3.6%]), *S pneumoniae* (4090 [2.5%]), and methicillin-resistant *Staphylococcus aureus* (MRSA) (3747 [2.3%]). Overall, 35 759 (22.1%) patients had a laboratory-identified etiology (19.4% bacterial, 3.2% viral, and 0.1% fungi). Table 2 lists *ICD-9* codes and laboratory results. The proportions of patients with positive laboratory findings and with organism-specific positive *ICD-9* codes were, respectively, 5.4% and 0.8% for methicillin-susceptible *S aureus* (MSSA), 3.6% and 2.3% for MRSA, 2.0% and 0.4% for *Escherichia coli*, 1.3% and 0.6% for *Klebsiella pneumoniae*, 3.6% and 3.0% for *S pneumoniae*, and 2.7% and 1.6% for *Pseudomonas* species.

**Table 2.** Cross-Classification of Laboratory Findings by *ICD-9* Diagnosis Code for 12 Specific Pneumonia Microbial Agents

Organism	No. (%)			
	*ICD-9*+/laboratory+	*ICD-9*−/ laboratory−	*ICD-9*+/ laboratory−	*ICD-9*−/laboratory+
MSSA	1233 (0.8)	152 722 (94.5)	120 (0.1)	7454 (4.6)
MRSA	2849 (1.8)	154 379 (95.6)	898 (0.6)	2936 (1.8)
*Streptococcus pneumoniae*	3525 (2.2)	154 668 (95.8)	1280 (0.8)	2345 (1.5)
*Pseudomonas* spp	2052 (1.3)	156 651 (97.0)	505 (0.3)	2321 (1.4)
*Escherichia coli*	557 (0.3)	158 240 (98.0)	71 (0.0)	2661 (1.6)
*Klebsiella pneumoniae*	774 (0.5)	159 154 (98.5)	205 (0.1)	1396 (0.9)
*Haemophilus influenzae*	708 (0.4)	159 747 (98.9)	128 (0.1)	946 (0.6)
*Mycoplasma pneumoniae*	564 (0.3)	160 126 (99.1)	348 (0.2)	491 (0.3)
*Legionella* spp	486 (0.3)	160 806 (99.6)	103 (0.1)	134 (0.1)
Influenza virus	4168 (2.6)	155 460 (96.2)	1723 (1.1)	178 (0.1)
Respiratory syncytial virus	41 (0.0)	161 339 (99.9)	20 (0.0)	129 (0.1)
Parainfluenza virus	20 (0.0)	161 371 (99.9)	15 (0.0)	123 (0.1)

Abbreviations: *ICD-9*, *International Classification of Diseases, Ninth Revision*; *ICD-9*+, presence of an *ICD-9* code; *ICD-9*−, absence of an ICD-9 code; laboratory+, presence of a laboratory sample identifying an organism; laboratory−, absence of a laboratory sample identifying an organism; MSSA, methicillin-sensitive *Staphylococcus aureus*; MRSA, methicillin-resistant *Staphylococcus aureus*; spp, several species.

Among 34 263 (21.2%) patients with either an organism-specific code or laboratory evidence, concordance between the 2 existed in fewer than half (eFigure in the Supplement). Table 3 shows the characteristics of *ICD-9* coding against the microbiology criterion standard for certain common organisms. In general, specificities were high, with 98.9% for influenza virus and 99.9% for MSSA. Sensitivities were substantially lower for most organisms, ranging from 95.9% (95% CI, 95.3%-96.5%) for influenza virus to 14.0% (95% CI, 8.8%-20.8%) for parainfluenza virus. Although both NPV and PPV values were higher than 75% for most bacterial organisms, owing to the low prevalence, the NPVs were substantially higher than the PPVs. The PPVs varied widely, from as low as 57.1% (95% CI, 39.4%-73.7%) for parainfluenza virus to as high as 91.1% (95% CI, 89.5%-92.6%) for MSSA, and were notably lower (57.1%-70.8%) for mycoplasma (61.8%), influenza (70.8%), respiratory syncytial virus (67.2%), and parainfluenza virus (57.1%) than for most other organisms, eg, MRSA (76.0%), *E coli* (88.7%), and *Legionella* species (82.5%).

**Table 3.** Performance Measures of Organism-Specific *ICD-9* Pneumonia Diagnosis Codes for Identifying Organism-Positive Laboratory Findings

Organism	*ICD-9* code	*ICD-9* performance vs laboratory criterion standard, %^a^			
		Test performance		Predictive value	
		Sensitivity	Specificity	Positive	Negative
MSSA	482.41	14.2	99.9	91.1	95.4
MRSA	482.42	49.3	99.4	76.0	98.1
*Streptococcus pneumoniae*	481, 482.30	60.1	99.2	73.4	98.5
*Pseudomonas* spp	482.1	46.9	99.7	80.3	98.5
*Escherichia coli*	482.82	17.3	100	88.7	98.4
*Klebsiella pneumoniae*	482	35.7	99.9	79.1	99.1
*Haemophilus influenzae*	482.2	42.8	99.9	84.7	99.4
*Mycoplasma pneumoniae*	483	53.5	99.8	61.8	99.7
*Legionella* spp	482.84	78.4	99.9	82.5	99.9
Influenza virus	487.x, 488.x	95.9	98.9	70.8	99.9
Respiratory syncytial virus	480.1	24.1	100	67.2	99.9
Parainfluenza virus	480.2	14.0	100	57.1	99.9

Abbreviations: *ICD-9*, *International Classification of Diseases, Ninth Revision*; MRSA, methicillin-resistant *Staphylococcus aureus*; MSSA, methicillin-sensitive *Staphylococcus aureus*; spp several species*.*

^a^ Microbiological data (test results for blood or respiratory culture, urinary antigen, or polymerase chain reaction) are considered as the criterion standard.

Temporal trends for 5 selected organisms are given in Table 4. Despite year-to-year variance, there do not appear to be consistent trends in sensitivity, specificity, or PPVs.

**Table 4.** Temporal Trends of *ICD-9* and Laboratory Data for Selected Organisms

Organism	Performance	%					
		Overall (n = 161 529)	July 2010-June 2011 (n = 37 259)	July 2011-June 2012 (n = 31 302)	July 2012-June 2013 (n = 33 803)	July 2013-June 2014 (n = 32 388)	July 2014-June 2015 (n = 30 148)
MSSA	*ICD-9*+	0.8	0.8	0.8	0.8	0.9	0.9
	Laboratory+	5.4	5.8	5.4	5.4	5.3	4.8
	Sensitivity	14.2	13.4	12.6	13.4	15.7	16.4
	Specificity	99.9	99.9	99.9	99.9	99.9	99.9
	PPV	91.1	92.8	91.3	89.3	90.7	91.4
	NPV	95.4	94.9	95.2	95.3	95.5	95.9
MRSA	*ICD-9*+	2.3	2.4	2.4	2.4	2.4	2.1
	Laboratory+	3.6	4.0	3.7	3.5	3.4	3.2
	Sensitivity	49.3	47.1	48.9	50.3	50.7	49.9
	Specificity	99.4	99.5	99.4	99.4	99.3	99.5
	PPV	76.0	80.3	75.7	75.6	72.4	75.5
	NPV	98.1	97.8	98.1	98.2	98.3	98.4
*Streptococcus pneumoniae*	*ICD-9*+	3.0	3.2	2.8	3.3	2.9	2.6
	Laboratory+	3.6	3.9	3.5	4.0	3.5	3.1
	Sensitivity	60.1	61.8	60.2	61.3	58.7	56.9
	Specificity	99.2	99.2	99.3	99.1	99.1	99.2
	PPV	73.4	75.5	74.9	74.3	70.8	70.1
	NPV	98.5	98.5	98.6	98.4	98.5	98.6
*Pseudomonas* spp	*ICD-9*+	1.6	1.8	1.7	1.6	1.4	1.3
	Laboratory+	2.7	3.0	3.0	2.7	2.5	2.3
	Sensitivity	46.9	49.5	44.5	48.3	45.9	45.5
	Specificity	99.7	99.6	99.7	99.7	99.7	99.7
	PPV	80.3	81.0	79.4	79.8	80.8	80.1
	NPV	98.5	98.5	98.3	98.6	98.6	98.7
Influenza virus	*ICD-9*+	3.7	1.9	0.9	4.1	4.7	6.9
	Laboratory+	2.7	1.3	0.6	3.1	3.3	5.4
	Sensitivity	95.9	91.2	92.9	96.0	96.9	96.9
	Specificity	98.9	99.3	99.7	98.8	98.4	98.3
	PPV	70.8	61.3	63.8	72.2	67.4	76.4
	NPV	99.9	99.9	100	99.9	99.9	99.8

Abbreviations: *ICD-9*, *International Classification of Diseases, Ninth Revision*; *ICD-9*+, presence of an *ICD-9* code; laboratory+, presence of a laboratory sample identifying an organism; MRSA, methicillin-resistant *Staphylococcus aureus*; MSSA, methicillin-sensitive *Staphylococcus aureus*; PPV, positive predictive value; NPV, negative predictive value; spp, several species.

## Discussion

In this cross-sectional diagnostic study of more than 160 000 patients undergoing culture or antigen testing for pneumonia infection in 178 US hospitals, we found that just 35 759 (22.1%) had an identified pathogen. *ICD-9*-coded organisms and laboratory findings differed notably. Although specificities and NPVs exceeded 95% for all codes, sensitivities ranged from 95.9% for influenza virus to 14.0% for parainfluenza virus, and PPVs were as high as 91.1% (95% CI, 89.5%-92.6%) for *S aureus* and as low as 57.1% (95% CI, 39.4%-73.7%) for parainfluenza virus. Because of the high specificities, for most diagnoses an *ICD-9* code was a reliable marker of a positive culture, but because of the low sensitivities, use of only administrative codes may potentially undercount almost all diagnoses.

Previous studies have examined the concordance between administrative and clinical data in various infectious syndromes, including pneumonia. Guevara and colleagues^9^ reported sensitivity of 58.3% for the pneumococcal pneumonia code (481.0), similar to what we observed. Schweizer et al^10^ questioned the validity of the *ICD-9* code for identifying incident MRSA infection ([V09] not limited to pneumonia), and found sensitivity of 24% with a PPV of 31%, lower than our findings but using a different coding approach. In a study focused on multidrug-resistant organisms, Burnham and colleagues^11^ found that a higher rate of coding for MRSA was associated with infectious disease consultation, and counseled against using that *ICD-9* code to estimate rates of multidrug-resistant organism infection in hospitals. The present study expands on this body of validation work by increasing the pool of common pneumonia pathogens beyond *S pneumoniae* and MRSA.

Understanding the epidemiology of pneumonia is important for resource allocation and risk prediction based on demographic characteristics and geospatial location.^12^ The low sensitivity of administrative data with regard to specific microorganisms has implications for interpreting epidemiological studies. Smith and colleagues,^13^ for example, used the Nationwide Inpatient Sample to explore the association between introduction of pneumococcal vaccine and distribution of pathogens among admissions of patients with pneumonia. They reported a reduction in *S pneumoniae* among pneumonia codes following the year 2000, suggesting that the pneumococcal vaccination was conferring its desired benefits. Our findings that the *S pneumoniae ICD-9* code identifies only 54% of culture-confirmed infections might call such an assertion into question. However, if coding practices remained constant throughout the study time frame, relative reductions in longitudinal trends would be unaffected by such discrepancies. We found that over a 5-year period sensitivity of the *S pneumoniae* code declined slightly, while specificity remained constant.

The high specificities of administrative codes for individual uncommon organisms make administrative data well suited for deriving predictive models, because specificity is a primary component of PPV when prevalence is low. PPV exceeded 70% for influenza and all bacterial species except mycoplasma, suggesting that characteristics of patients who have codes for specific organisms may be representative of patients who actually have infection with those organisms. At least 9 models have been created to predict drug-resistant organisms, such MRSA and *Pseudomonas* species*,* in pneumonia. Nearly all models perform better than the health care–associated pneumonia criteria, but no single model is yet accurate enough to guide antibiotic stewardship.^14,15^ More sophisticated models would be particularly useful as decision aids to help clinicians optimize empirical treatment while avoiding overuse of broad-spectrum agents. High specificity is critical to ensure the accuracy of such predictive instruments. Although missing cases may have minimal implications for the model’s discrimination, low sensitivity of *ICD-9* data could result in miscalibration, consistently underpredicting risk.

Similarly, changing resistance patterns over time are of concern to clinicians prescribing empirical antibiotic therapy. The Centers for Disease Control and Prevention National Healthcare Safety Network provides robust data describing trends in resistance, for example, changing prevalence of MRSA and the emergence of multidrug-resistant gram-negative bacteria.^16^ Our temporal analysis shows that the association between administrative and laboratory data for MSSA and MRSA has been stable between 2010 and 2014, with a slight uptick in sensitivity in 2015 for both organisms. This finding suggests utility for efforts at more generalizable infection surveillance using administrative data.

### Limitations

This study has limitations. The case selection algorithm may have insufficiently discriminated pneumonia from other infection diagnoses, and identified pathogens may represent colonization rather than infection. For example, many patients had culture growth but no corresponding *ICD-9* coding event. This subset of patients tended to have more comorbid conditions. Although all patients had a diagnosis of pneumonia, it is possible that the specific pneumonia code was missed owing to truncation of diagnoses. MRSA and MSSA may also be coded as present based on the result of a nasal swab, which we did not include because nasal passages may not accurately represent lung flora. The presence of an *ICD-9* code indicating infection without culture results could represent coding based on clinical suspicion, available data from a transferring institution, or late reporting of growth after hospital discharge. We also excluded patients who did not have any cultures. Although this number was small, it may have introduced a bias in detection rates.

Of note, *International Statistical Classification of Diseases and Related Health Problems, Tenth Revision (ICD-10)* coding was implemented in the US on October 1, 2015, and is far more detailed than *ICD-9*, making it difficult to study a population across the transition. Thus, our analysis is limited to 2010-2015. Nonetheless, the process by which codes are chosen has not changed, and it is possible to crosswalk from *ICD-10* to *ICD-9*. In addition, it is not known how our results may be applicable to other large administrative data sets. Because this was a national sample, coding is likely to be similar, but it is possible that hospitals outside of the Premier Healthcare Database have different coding patterns. Validation in additional data sets would be welcome.

## Conclusions

In this study, organism-specific administrative codes in hospitalized patients undergoing laboratory testing for infection appear to have limited sensitivities in the setting of pneumonia, although specificities and NPVs are high, and PPVs are reasonable considering the low pretest probabilities and consequent challenges of ruling in specific organisms. This finding may have important implications for the reliability of research conducted in administrative databases. Although the high specificity is conducive to predictive modeling, low sensitivities may limit the utility of organism-specific administrative codes for surveillance purposes, as organism-specific prevalence estimates based on administrative codes may underestimate true organism-specific burden. Future studies may need to examine whether microbiology trends indicated by *ICD-9* codes represent actual pathogen shifts or are consequences of alterations in coding practices.

## Notes

Supplement. **eFigure.** Venn Diagram of Etiology and Laboratory Result **eTable.** Tests Required for Study Inclusion
