# Supplementary material for: Assessment of the Accuracy of Using ICD-9 Diagnosis Codes to Identify Pneumonia Etiology in Patients Hospitalized With Pneumonia
Source: JAMA Netw Open. 2020 Jul 22;3(7):e207750. doi: 10.1001/jamanetworkopen.2020.7750 (PMC7376393; doi:10.1001/jamanetworkopen.2020.7750)
Supplement: Supplement. — eFigure. Venn Diagram of Etiology and Laboratory Result eTable. Tests Required for Study Inclusion [file jamanetwopen-3-e207750-s001.pdf]

Supplementary Online Content

Higgins TL, Deshpande A, Zilberberg MD, et al. Assessment of the accuracy of using *ICD-9* diagnosis codes to identify pneumonia etiology in patients hospitalized with pneumonia. *JAMA Netw Open*. 2020;3(7):e207750. doi:10.1001/jamanetworkopen.2020.7750

eFigure. Venn Diagram of Etiology and Laboratory Result

eTable. Tests Required for Study Inclusion

This supplementary material has been provided by the authors to give readers additional information about their work.

© 2020 Higgins TL et al. *JAMA Network Open.*

eFigure. Venn Diagram of Etiology and Laboratory Result

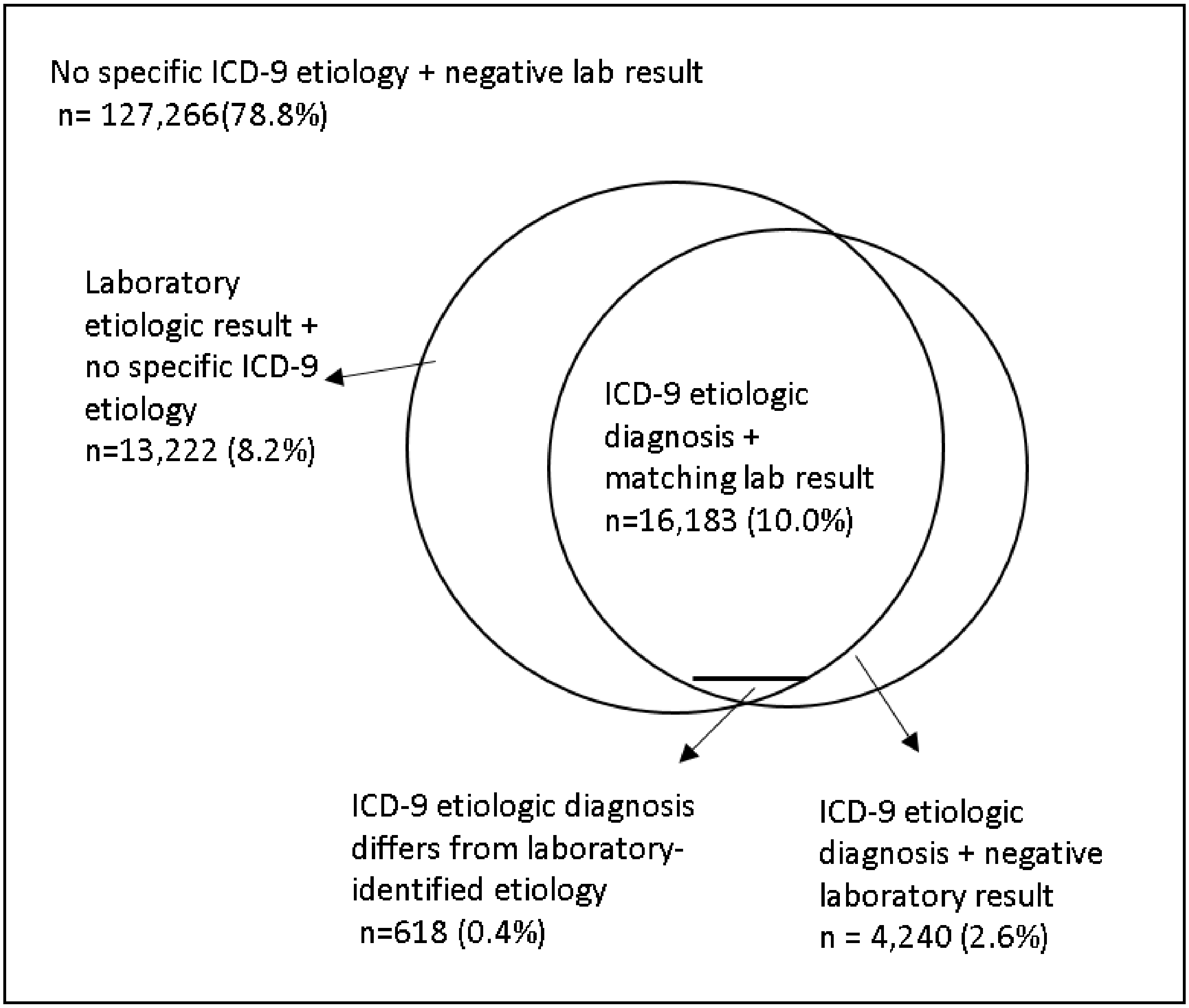

© 2020 Higgins TL et al. *JAMA Network Open.*

eTable. Tests Required for Study Inclusion

Factor	Total (N=161,529)
dx_486only, No. (%)	107,601(66.6)
480.0: Pneumonia due to adenovirus, No. (%)	4(0.00)
480.1: Pneumonia due to respiratory syncytial virus, No. (%)	61(0.04)
480.2: Pneumonia due to parainfluenza virus, No. (%)	35(0.02)
480.3: Pneumonia due to SARS-associated coronavirus, No. (%)	1(0.00)
480.8: Pneumonia due to other virus not elsewhere classified, No. (%)	130(0.08)
480.9: Viral pneumonia, unspecified, No. (%)	243(0.15)
481: Pneumococcal pneumonia, No. (%)	4,090(2.5)
482.0: Pneumonia due to Klebsiella pneumoniae, No. (%)	979(0.61)
482.1: Pneumonia due to Pseudomonas, No. (%)	2,557(1.6)
482.2: Pneumonia due to Hemophilus influenzae, No. (%)	836(0.52)
482.30: Pneumonia due to Streptococcus, unspecified, No. (%)	729(0.45)
482.31: Pneumonia due to Streptococcus, group A, No. (%)	96(0.06)
482.32: Pneumonia due to Streptococcus, group B, No. (%)	142(0.09)
482.39: Pneumonia due to other Streptococcus, No. (%)	268(0.17)
482.40: Pneumonia due to Staphylococcus, unspecified, No. (%)	176(0.11)
482.41: Methicillin susceptible pneumonia - Staphylococcus aureus, No. (%)	1,353(0.84)
482.42: Methicillin resistant pneumonia - Staphylococcus aureus, No. (%)	3,747(2.3)
482.49: Other Staphylococcus pneumonia, No. (%)	87(0.05)
482.81: Pneumonia due to anaerobes, No. (%)	89(0.06)
482.82: Pneumonia due to escherichia coli, No. (%)	628(0.39)
482.83: Pneumonia due to other gram-negative bacteria, No. (%)	3,352(2.1)
482.84: Pneumonia due to Legionnaires disease, No. (%)	589(0.36)
482.89: Pneumonia due to other specified bacteria, No. (%)	97(0.06)
482.9: Bacterial pneumonia, unspecified, No. (%)	3,792(2.3)
483.0: Pneumonia due to mycoplasma pneumoniae, No. (%)	912(0.56)
483.1: Pneumonia due to chlamydia, No. (%)	22(0.01)
483.8: Pneumonia due to other specified organism, No. (%)	162(0.10)
484.1: Pneumonia in cytomegalic inclusion disease, No. (%)	16(0.01)
484.3: Pneumonia in whooping cough, No. (%)	3(0.00)
484.5: Pneumonia in anthrax, No. (%)	0(0.0)
484.6: Pneumonia in aspergillosis, No. (%)	96(0.06)
484.7: Pneumonia in other systemic mycoses, No. (%)	42(0.03)
484.8: Pneumonia in other infectious diseases classified elsewhere, No. (%)	12(0.01)
485: Bronchopneumonia, organism unspecified, No. (%)	728(0.45)
486: Pneumonia, organism unspecified, No. (%)	110,360(68.3)
Pneumonia due to influenza (487.x 488.x), No. (%)	5,891(3.6)
487.0: Influenza with pneumonia, No. (%)	3,670(2.3)
487.1: Influenza with other respiratory manifestations, No. (%)	976(0.60)
487.8: Influenza with other manifestations, No. (%)	118(0.07)

© 2020 Higgins TL et al. *JAMA Network Open.*

488.01: Influenza due to identified avian influenza virus with pneumonia, No. (%)	64(0.04)
488.02: Influenza due to identified avian influenza virus with other respiratory manifestations, No. (%)	111(0.07)
488.09: Influenza due to identified avian influenza virus with other manifestations, No. (%)	9(0.01)
488.11: Influenza due to identified 2009 H1N1 influenza virus with pneumonia, No. (%)	177(0.11)
488.12: Influenza due to identified 2009 H1N1 influenza virus respiratory manifestations, No. (%)	100(0.06)
488.19: Influenza due to identified 2009 H1N1 influenza virus with other manifestations, No. (%)	10(0.01)
488.81: Influenza due to identified novel influenza A virus with pneumonia, No. (%)	299(0.19)
488.82: Influenza due to identified novel influenza A virus respiratory manifestations, No. (%)	421(0.26)
488.89: Influenza due to identified novel influenza A virus with other manifestations, No. (%)	45(0.03)
507.0: Pneumonitis due to inhalation of food or vomitus, No. (%)	27,306(16.9)

© 2020 Higgins TL et al. *JAMA Network Open.*
